# Effect of Body Weight on Glycaemic Indices in People with Type 1 Diabetes Using Continuous Glucose Monitoring

**DOI:** 10.3390/jcm13175303

**Published:** 2024-09-07

**Authors:** Maria A. Christou, Panagiota A. Christou, Daphne N. Katsarou, Eleni I. Georga, Christos Kyriakopoulos, Georgios Markozannes, Georgios A. Christou, Dimitrios I. Fotiadis, Stelios Tigas

**Affiliations:** 1 Department of Endocrinology, University Hospital of Ioannina, 45500 Ioannina, Greece; mariachristou1@gmail.com (M.A.C.); panagiotaxrist@hotmail.com (P.A.C.); georgios.christou@yahoo.gr (G.A.C.); 2 Department of Hygiene and Epidemiology, Faculty of Medicine, University of Ioannina, 45500 Ioannina, Greece; georgemarkozannes@gmail.com; 3 Unit of Medical Technology and Intelligent Information Systems, University of Ioannina, 45500 Ioannina, Greece; d.katsarou@uoi.gr (D.N.K.); egewrga@gmail.com (E.I.G.); fotiadis@uoi.gr (D.I.F.); 4 Department of Respiratory Medicine, University Hospital of Ioannina, 45500 Ioannina, Greece; ckyriako@yahoo.gr

**Keywords:** adiposity indices, body mass index, body weight, continuous glucose monitoring, glycaemic indices, obesity, overweight, type 1 diabetes, waist circumference

## Abstract

**Background/Objectives**: Obesity and overweight have become increasingly prevalent in different populations of people with type 1 diabetes (PwT1D). This study aimed to assess the effect of body weight on glycaemic indices in PwT1D. **Methods**: Adult PwT1D using continuous glucose monitoring (CGM) and followed up at a regional academic diabetes centre were included. Body weight, body mass index (BMI), waist circumference, glycated haemoglobin (HbA1c), and standard CGM glycaemic indices were recorded. Glycaemic indices were compared according to BMI, and correlation and linear regression analysis were performed to estimate the association between measures of adiposity and glycaemic indices. **Results**: A total of 73 PwT1D were included (48% normal weight, 33% overweight, and 19% obese). HbA1c was 7.2% (5.6–10), glucose management indicator (GMI) 6.9% (5.7–8.9), coefficient of variation (CV) for glucose 39.5% ± 6.4, mean glucose 148 (101–235) mg/dL, TIR (time in range, glucose 70–180 mg/dL) 66% (25–94), TBR_70_ (time below range, 54–69 mg/dL) 4% (0–16), TBR_54_ (<54 mg/dL) 1% (0–11), TAR_180_ (time above range, 181–250 mg/dL) 20% ± 7, and TAR_250_ (>250 mg/dL) 6% (0–40). Glycaemic indices and achievement (%) of optimal glycaemic targets were similar between normal weight, overweight, and obese patients. BMI was associated negatively with GMI, mean glucose, TAR_180_, and TAR_250_ and positively with TIR; waist circumference was negatively associated with TAR_250_. **Conclusions**: CGM-derived glycaemic indices were similar in overweight/obese and normal weight PwT1D. Body weight and BMI were positively associated with better glycaemic control. PwT1D should receive appropriate ongoing support to achieve optimal glycaemic targets whilst maintaining a healthy body weight.

## 1. Introduction

The prevalence of overweight and obesity in people with type 1 diabetes (PwT1D) is increasing at a rate similar to or higher compared to the general population. Specifically, data from the type 1 diabetes (T1D) Exchange Registry in the United States demonstrated that 29% of adults with T1D were overweight and 20% were obese [1]. Additionally, data from the international SWEET registry showed that 27% of girls and 22% of boys with T1D (age 2–18 years) were overweight or obese [2]. Temporal patterns in overweight and obesity show excessive weight gain in an adult T1D population of the EDC study [3]. Specifically, the prevalence of overweight and obesity increased from 29% and 3% to 42% and 23%, respectively, after 18 years of follow-up. Similarly, the prevalence of obesity in the DCCT/EDIC study increased from 2% to 28% after 12 years of follow-up [4].

There are several possible mechanisms of weight gain in PwT1D [5,6]. The most important cause is believed to be exposure of peripheral tissues to supraphysiologic insulin concentrations; subcutaneous insulin administration does not follow the natural route of insulin secretion into the portal circulation, bypasses the liver, and may cause peripheral fat accumulation. Intensive insulin therapy may increase insulin resistance leading to increased insulin dose, thus further promoting insulin-associated weight gain. Insulin therapy may also lead to alterations in the growth hormone—Insulin-like Growth Factor 1 (IGF1)—system which balances anabolism and catabolism and maintains body composition. Another possible explanation for weight gain in PwT1D is the increased hypoglycaemia risk associated with intensive insulin therapy and the consequent fear of hypoglycaemia which leads to compensatory carbohydrate intake when hypoglycaemia occurs, defensive snacking to avoid that, and abstinence from exercise. Other possible causes include genetic predisposition, duration of diabetes, and use of continuous subcutaneous insulin infusion (CSII) instead of multiple daily injections (MDIs) of insulin. Furthermore, excess body fat in PwT1D may lead to glucotoxicity and accelerate beta cell apoptosis, thus providing a possible explanation as a contributing factor for the increasing worldwide incidence of T1D in recent decades [7]. 

Importantly, overweight and obesity increase the risk for both diabetes-related and obesity-related complications in T1D patients, such as metabolic and cardiovascular disease, specific cancer types, poor mental health, and premature death [5]. An estimated 30–45% of patients with T1D have metabolic syndrome, and therefore up to half have “double diabetes”, a definition used for PwT1D who are overweight, and have a family history of type 2 diabetes and/or clinical features of insulin resistance [8]. Additionally, T1D patients who were registered in the Swedish National Diabetes registry and who were followed up for 11 years showed increased risk for major cardiovascular events, hospitalisations for heart failure, and cardiovascular and total mortality with increasing body mass index (BMI) [9]. These associations were more apparent in men than in women. Notably, T1D patients who display features of “double diabetes”, i.e., are overweight or obese, and have a family history of type 2 diabetes and/or clinical features of insulin resistance, are at higher risk of developing diabetes complications [8]. This happens independently of the level of glucose control, as assessed by glycated haemoglobin (HbA1c). 

The impact of adiposity on glycaemic control in youth and adults with T1D is unclear. Findings are conflicting with some studies showing a positive association between adiposity and better glycaemic control [10,11,12], while other studies demonstrated a negative association [13,14,15,16]. Therefore, in the present study, we aim to assess the effect of adiposity indices (body weight, BMI, and waist circumference) on glycaemic indices in adult PwT1D using continuous glucose monitoring (CGM).

## 2. Materials and Methods

This is a cross-sectional study conducted at the outpatient clinic of the Department of Endocrinology and Diabetes of the University Hospital of Ioannina, Greece from April 2023 to February 2024. Inclusion criteria were adult PwT1D on MDIs of insulin or CSII, using a CGM system and followed up at the outpatient clinic. Exclusion criteria were the following: age below 18 years, current pregnancy or breastfeeding, and acute systemic inflammation. 

All patients were evaluated by an experienced endocrinologist. A detailed medical history was obtained. The following data were recorded anonymously from the medical record: gender, age, diabetes duration, insulin delivery method (MDIs or CSII), CGM type, insulin type (long and fast acting), insulin units [insulin total daily dose (TDD) in 24 h and per kg of body weight], other medical history, and medications. A comprehensive physical examination was carried out. Body weight was measured with patients in light clothing and shoes removed. Height was measured using a wall-mounted height stadiometer. BMI was calculated as body weight/height^2^ (kg/m^2^). The patients’ waist circumference was measured at the midpoint between the last rib and the iliac crest. The percentage of normal weight (BMI 18.5–24.9 kg/m^2^), overweight (BMI 25.0–29.9 kg/m^2^), and obese (BMI ≥ 30 kg/m^2^) patients was recorded. Additionally, the percentage of males and females with a waist circumference >102 cm and >88 cm, respectively, according to the International Diabetes Federation (IDF) criteria for the diagnosis of abdominal obesity and metabolic syndrome, was estimated [17]. Haematological and biochemical parameters from fasting venous blood samples were evaluated at the hospital’s laboratory by standard methods as part of their routine assessment at the outpatient clinic.

The following CGM-derived metrics were recorded for the preceding 14 days: mean glucose, glucose management indicator (GMI), coefficient of variation (CV) for glucose, time in range (TIR, glucose 70–180 mg/dL), TBR_70_ (time below range, glucose 54–69 mg/dL), TBR_54_ (time below range, glucose < 54 mg/dL), TAR_180_ (time above range, glucose 181–250 mg/dL), and TAR_250_ (time above range, glucose > 250 mg/dL). The GMI is automatically calculated by each CGM device based on the following equation: GMI (%) = 3.31 + 0.02392 × (mean glucose in mg/dL), where mean glucose stands for the average sensor glucose concentration over the selected time period [18]. Achievement of therapeutic goals was recorded, in particular, the percentage of patients with HbA1c < 7%, GMI < 7%, CV ≤ 36%, TIR > 70%, TBR_70_ < 4%, TBR_54_ < 1%, TAR_180_ < 25%, and TAR_250_ < 5% [19,20].

All patients provided written informed consent. This study was performed in accordance with the Declaration of Helsinki and was approved by the Ethics Committee of the University Hospital of Ioannina (Institutional Review Board approval number 247/2020). 

Categorical variables were expressed as numbers (percentages), and continuous variables with or without normal distribution were expressed as mean ± standard deviation or median (range), respectively. A comparison of glycaemic indices according to BMI (normal weight, overweight, or obese) was performed. A chi-square test was employed to compare categorical variables. A two-tailed t-test or a Mann–Whitney test was performed to compare continuous variables with or without normal distribution, respectively. The association between measures of adiposity (body weight, BMI, and waist circumference) and glycaemic indices was assessed by Pearson or Spearman correlation analysis for parameters with or without normal distribution, respectively. Multiple linear regression analyses with adjustment for age, gender, diabetes duration, insulin delivery method, and insulin TDD/kg of body weight were employed to explore independent associations between adiposity and glycaemic indices. For 80% power and a one-sided 0.05 alpha, a total sample size of at least 48 patients was required to demonstrate a 20% difference in TIR between normal weight and obese patients, and at least 28 patients were required to demonstrate a 20% difference in CV for glucose. Statistical analyses were performed with the statistical programme Stata14. The statistical significance was set at a *p* value of <0.05.

## 3. Results

### 3.1. Patient Characteristics

Patient characteristics are shown in Table 1. Seventy-three consecutive patients with T1D, who attended the outpatient clinic, met the inclusion criteria, and agreed to take part in the study, were included. Out of those, 35 (48%) were normal weight, 24 (33%) overweight, and 14 (19%) obese. The median age was 39 (18–75) years, and 43 (59%) were males. Fifty-five (75%) patients received insulin treatment as MDIs, and eighteen (25%) used CSII. With respect to the type of CSII, 11 patients used MiniMed^TM^ 640G (Medtronic, Minneapolis, MN, USA), 6 patients MiniMed^TM^ 780G (Medtronic, Minneapolis, MN, USA), and 1 patient Accu-Chek^®^ Combo (Roche, Basel, Switzerland). Regarding the type of CGM system, 52 (71%) patients used the FreeStyle Libre (Abbott, Chicago, IL, USA; 48 used the 1st generation and 4 used the 2nd generation system), 15 (21%) the Guardian^TM^ Sensor (Medtronic, Minneapolis, MN, USA) as part of a combined pump–CGM system (9 patients used Sensor 3 and 6 patients used Sensor 4), and 6 (8%) the GlucoMen^®^ Day CGM system (Menarini, Florence, Italy). Diabetes duration was 19 ± 11 years, and insulin TDD/kg of body weight was 0.63 ± 0.19 units/kg. Age, gender distribution, diabetes duration, insulin delivery method, and insulin TDD were similar in normal weight, overweight, and obese patient groups (*p* = ns). No differences were observed in patient characteristics according to BMI status. Notably, body weight, BMI, and waist circumference were similar in patients who received MDIs or used CSII: 75 ± 17 kg versus 81 ± 21 kg (*p* value = 0.196), 25.7 ± 4.9 kg/m^2^ versus 27.7 ± 6.8 kg/m^2^ (*p* value = 0.175), and 88 ± 15 cm versus 94 ± 20 cm (*p* value = 0.228), respectively.

### 3.2. Measures of Adiposity

Body weight was 77 ± 18 kg, BMI 25.6 (18.5–43.3) kg/m^2^, waist circumference in males 94 ± 14 cm, and waist circumference in females 76 (64–128) cm. Waist circumference was >102 cm in 10 (24%) male patients and >88 cm in 7 (23%) female patients. As expected, adiposity indices were significantly higher in overweight and obese patients compared to normal weight patients. Adiposity indices are presented in Table 2.

### 3.3. Glycaemic Indices and Achievement of Optimal Glycaemic Targets

HbA1c in the total study population was 7.2% (5.6–10) and CGM-derived glycaemic indices were as follows: GMI 6.9% (5.7–8.9), CV 39.5% ± 6.4, mean glucose 148 (101–235) mg/dL, TIR 66% (25–94), TBR_70_ 4% (0–16), TBR_54_ 1% (0–11), TAR_180_ 20% ± 7, and TAR_250_ 6% (0–40). The glycaemic indices did not differ between normal weight, overweight, and obese patients with the exception of TAR_250_ which was significantly lower in obese patients compared to normal weight patients: 5% ± 4 versus 7% (0–40), respectively (*p* value = 0.026). Additionally, GMI and mean glucose were marginally lower in obese patients compared to normal weight patients: 6.7% ± 0.4 versus 6.9% (6.0–8.9) (*p* value = 0.072) and 142 ± 18 mg/dL versus 151 (113–235) mg/dL (*p* value = 0.063), respectively. Regarding percentage of achievement of optimal glycaemic targets in the total study population, HbA1c < 7% and GMI < 7% were observed in 26 (36%) and 41 (57%) patients, respectively, CV ≤ 36% in 25 (34%), TIR > 70% in 22 (30%), TBR_70_ < 4% in 28 (38%), TBR_54_ < 1% in 34 (47%), TAR_180_ < 25% in 55 (75%), and TAR_250_ < 5% in 26 (36%). Achievement (%) of optimal glycaemic targets was similar between normal weight, overweight, and obese patients. Glycaemic indices and percentage of achievement of optimal glycaemic targets are presented in Table 3 and Table 4, respectively.

### 3.4. Associations between BMI, Body Weight, Waist Circumference, and Glycaemic Indices

BMI was negatively correlated with GMI (r = −0.227, *p* value = 0.049), mean glucose (r = −0.231, *p* value = 0.049), and TAR_250_ (r = −0.251, *p* value = 0.033) in the total study population. Similarly, body weight was negatively correlated with GMI (r = −0.225, *p* value = 0.049), mean glucose (r = −0.225, *p* value = 0.049), and TAR_250_ (r = −0.252, *p* value = 0.032). However, the strength of the above correlations was weak. Waist circumference was not correlated with glycaemic indices. Correlations between BMI, body weight, waist circumference, and glycaemic indices are shown in Table 5.

### 3.5. Effect of BMI, Body Weight, and Waist Circumference on Glycaemic Indices

In the linear regression analysis, BMI was negatively associated with GMI (β = −0.035, *p* value = 0.012), mean glucose (β = −1.517, *p* value = 0.009), TAR_180_ (β = −0.302, *p* value = 0.047) and TAR_250_ (β = −0.516, *p* value = 0.014) and positively associated with TIR (β = 0.636, *p* value = 0.039) in the total population. Moreover, body weight was negatively associated with GMI (β = −0.010, *p* value = 0.035), mean glucose (β = −0.427, *p* value = 0.028), and TAR_250_ (β = −0.155, *p* value = 0.027). Additionally, body weight was positively associated with TIR marginally without reaching statistical significance (β = 0.188, *p* value = 0.067). In the linear regression analysis, waist circumference was negatively associated with TAR_250_ (β = −0.142, *p* value = 0.049). The results from the linear regression analysis are presented in Table 6.

## 4. Discussion

Over half of the patients with T1D in the present study were overweight or obese with a significant percentage (24% of males and 23% of females) presenting with central obesity. No differences were observed in age, diabetes duration, insulin dose, and insulin delivery method (MDIs or CSII) between normal weight, overweight, and obese patients. HbA1c and the other CGM-derived glycaemic indices did not differ between patients according to BMI status except for TAR_250,_ and marginally for GMI and mean glucose, which were significantly lower in obese patients compared to normal weight patients. Body weight, BMI, and waist circumference were positively associated with better glycaemic control. Achievement of optimal glycaemic targets was similar between normal weight, overweight and obese patients. However, a significant proportion of T1D patients did not reach the therapeutic goals for glycaemic control. 

The association between body weight and glycaemic control is complex, and research findings on this subject have been controversial. In accordance with our findings, a positive association between adiposity measures and improved glycaemic profile was reported by Nansel et al. [10]. The investigators examined associations of BMI with HbA1c in youth with T1D participating in a 2-year intervention study targeting family diabetes management and demonstrated that BMI was inversely related to HbA1c. Furthermore, Williams et al. compared the prevalence and incidence of overweight in the EDC cohort with those in the general population (NHANES) over 6.9 years [11]. The authors found that subjects who had a major improvement in glycaemic control (>2% decrease in HbA1c) gained more weight (8.2 ± 6.8 kg versus 2.7 ± 6.0 kg, *p* value < 0.001) and had a greater risk of being overweight (25.6% versus 10.8%, *p* value < 0.01) compared to those with no major improvement in glycaemia. In the EURODIAB study, Ferriss et al. examined 3250 PwT1D (age 15–60 years) at baseline and 7.3 years later and observed that the HbA1c change from baseline to follow-up was more favourable in those who gained 5 kg or more than in patients who gained less or no weight or lost weight [12]. 

On the other hand, some studies demonstrated a negative association between adiposity and better glycaemic control. Specifically, in an 18-month randomised controlled dietary intervention trial of youth with T1D, 1,5-Anhydroglucitol, which is sensitive to recent glucose excursions and decreases during hyperglycaemia, was inversely associated with BMI, and dual-energy X-ray absorptiometry-derived %fat, total fat mass, and trunk %fat were positively associated with %glucose > 180 mg/dL and >126 mg/dL derived from a 3-day CGM system [iPro^TM^ (Medtronic, Minneapolis, MN, USA)] [13]. During the DCCT study, patients with T1D were randomly assigned to intensive or conventional diabetes treatment and underwent intima-media thickness and coronary artery calcium score measurements during follow-up in the EDIC study [14]. The study revealed that at both one and six years of follow-up, HbA1c was higher in excess weight gainers (those who belonged in the fourth quartile of BMI and whose BMI increased by at least 4.39 kg/m^2^) compared to minimal gainers (those who belonged in the three first quartiles of BMI). Additionally, Flokas et al. aimed to identify modifiable behavioural characteristics of overweight and obese patients with T1D from the T1D Exchange Registry who achieve optimal glycaemic control (mean most recent HbA1c ≤ 7.5%) [15]. In the optimally controlled cohort, 27% of subjects were overweight or obese versus 30% in the suboptimally controlled cohort. Moreover, Lee et al. investigated the association between BMI and average HbA1c levels in T1D patients over 18 months [16]. An inverse association of BMI with HbA1c levels was observed in the low BMI group (<21 kg/m^2^), while a positive association was shown in the high BMI group (≥23 kg/m^2^). Interestingly, recent evidence suggests that in genetically at-risk children, a higher total energy intake and BMI may influence the development of autoimmunity and progression to clinical T1D [21,22,23].

The observed conflicting findings regarding the association between adiposity indices and glycaemic control in T1D patients may be attributed to the study of different populations and the use of different designs and analytic methods in the respective studies. A recent analysis of a T1D population of the SEARCH study cohort concluded that there are distinct subgroups of youth and young adults with T1D that share weight–glycaemia phenotypes in need of tailored interventions [24]. A low BMI in T1D is commonly associated with a catabolic state due to poor glycaemic control. Perhaps not surprisingly, a low BMI was an independent risk factor for all-cause mortality in 725 African Americans after 2 years of follow-up [25]. Moreover, the relationship of adiposity with mortality in T1D patients after 20 years of follow-up in the EDC study exhibited a U-shaped relationship like in the general population, albeit with a markedly increased risk in those who were underweight [26].

Several studies have demonstrated that higher insulin doses and intensive insulin therapy can result in accelerated weight gain [3,12,27,28,29]. Theoretically, intensive insulin therapy and the subsequent hyperinsulinemia may suppress lipolysis, shift fuel use from fatty acids to glucose, decrease glycosuria and the related caloric loss, decrease daily energy expenditure, increase hunger and food intake, and therefore increase body weight and fat mass [30,31,32,33]. Furthermore, research observations and clinical experience suggest that the use of CSII compared to MDIs may promote weight gain [34,35]. However, some other studies found no such difference in weight gain between CSII and MDI-treated patients [36,37,38,39,40]. In our study, both the total daily insulin dose and the insulin delivery method were similar between normal weight, overweight, and obese patients. Moreover, the studied adiposity indices did not differ based on the insulin delivery method (CSII versus MDIs). Our finding of a positive association between BMI and waist circumference with better glycaemic control seems paradoxical. Given that all our study participants were using CGM, and a significant portion were also using insulin pumps, it is possible that the confidence and dietary flexibility associated with the use of advanced technology led to increased caloric intake and weight gain, as well as improved glycaemic control [39,41]. Indeed, CGM use has been shown to significantly improve glycaemic control in both MDIs as well as insulin pump users [1,40,42].

The main strength of the present study is the fact that all patients used a CGM system for a considerable time period allowing for the extraction of robust data about glycaemic indices. Our study has certain limitations. Due to the cross-sectional study design, a causative association between the effect of adiposity indices on glycaemic indices cannot be established. Additionally, body composition measurement was not performed and therefore muscle mass and body fat percentage were not assessed. Moreover, data regarding diet and physical activity were not recorded. Patients also used different types of CGM systems (intermittently scanned or real-time continuous glucose monitoring) and different types of insulin delivery methods (MDIs or CSII). Further studies addressing the above points should be performed.

Therapeutic interventions in T1D patients should aim towards the achievement of optimal glycaemic targets. A multidisciplinary approach is also required for obesity management, as well as the implementation of personalised and targeted interventions that include lifestyle changes (healthy diet, regular exercise, and smoking and alcohol abstinence) and behavioural modifications. Management of blood pressure, lipids, and thrombosis risk is necessary to reduce cardiovascular risk. Finally, there is evidence suggesting that pharmacological agents used to manage glycaemic control, insulin resistance, and obesity in type 2 diabetes, such as metformin, glucagon-like peptide 1 receptor agonists (GLP-1 RAs), and sodium-glucose cotransporter 2 inhibitors (SGLT2is), may have benefits as adjunctive to insulin therapy in obese PwT1D [6,43,44].

In conclusion, body weight and BMI were positively associated with better glycaemic control in our study population. CGM-derived glycaemic indices were similar in overweight/obese and normal weight PwT1D. The use of advanced diabetes technology may result in weight gain by allowing for dietary flexibility without unfavourably affecting glycaemic control. Measures to prevent and manage the increasing prevalence of obesity in PwT1D are needed.

## Figures and Tables

**Table 1:** Patient characteristics.

	Total Population	Normal Weight	Overweight	Obese	Overweight/ Obese	*p* Value ^1^	*p* Value ^2^	*p* Value ^3^
**Number of patients**	73	35	24	14	38	-	-	-
**Male**	43 (59%)	17 (49%)	17 (71%)	9 (64%)	26 (68%)	0.319	0.089	0.085
**Age (years)**	39 (18–75)	38 (18–75)	41 ± 13	45 ± 11	42 ± 12	0.147	0.354	0.252
**Insulin delivery method**						0.357	0.854	0.732
MDIs	55 (75%)	27 (77%)	19 (79%)	9 (64%)	28 (74%)			
CSII	18 (25%)	8 (23%)	5 (21%)	5 (36%)	10 (26%)			
**CGM system**						0.127	0.914	0.543
Abbott FreeStyle Libre	52 (71%)	27 (77%)	18 (75%)	7 (50%)	25 (66%)			
Menarini GlucoMen^®^ Day	6 (8%)	2 (6%)	1 (4%)	3 (21%)	4 (11%)			
Medtronic GuardianTM Sensor	15 (21%)	6 (17%)	5 (21%)	4 (29%)	9 (24%)			
**Diabetes duration (years)**	19 ± 11	18 ± 12	18 ± 12	24 ± 7	20 ± 11	0.101	0.908	0.486
**Insulin TDD/kg (U/kg)**	0.63 ± 0.19	0.61 ± 0.21	0.64 ± 0.19	0.65 ± 0.16	0.63 (0.36–0.92)	0.493	0.523	0.426

**Abbreviations:** CGM: continuous glucose monitoring; CSII: continuous subcutaneous insulin infusion; MDIs: multiple daily injections; TDD: total daily dose; U: units. **Notes:** Categorical variables are expressed as numbers (percentages), and continuous variables with or without normal distribution are expressed as mean ± standard deviation or median (range), respectively. ^1^ Comparison between normal weight and obese patients. ^2^ Comparison between normal weight and overweight patients. ^3^ Comparison between normal weight and overweight/obese patients.

**Table 2:** Adiposity indices.

	Total Population	Normal Weight	Overweight	Obese	Overweight/ Obese	*p* Value ^1^	*p* Value ^2^	*p* Value ^3^
**Body weight (kg)**	77 ± 18	63 ± 10	82 ± 8	101 ± 13	89 ± 14	<0.001	<0.001	<0.001
**BMI (kg/m^2^)**	25.6 (18.5–43.3)	22.4 (18.5–24.7)	27.1 ± 1.3	35.0 ± 4.3	28.5 (25.5–43.3)	<0.001	<0.001	<0.001
**WC_male_ (cm)**	94 ± 14	82 (63–91)	95 ± 7	114 ± 10	102 ± 12	<0.001	<0.001	<0.001
**WC_male_ > 102 cm**	10 (24%)	0 (0%)	2 (13%)	8 (89%)	10 (40%)	<0.001	0.133	0.003
**WC_female_ (cm)**	76 (64–128)	70 (64–88)	88 ± 15	115 ± 10	99 ± 19	0.001	0.014	0.001
**WC_female_ > 88 cm**	7 (23%)	0 (0%)	2 (29%)	5 (100%)	7 (58%)	<0.001	0.018	<0.001

**Abbreviations:** BMI: body mass index; WC: waist circumference. **Notes:** Categorical variables are expressed as numbers (percentages), and continuous variables with or without normal distribution are expressed as mean ± standard deviation or median (range), respectively. ^1^ Comparison between normal weight and obese patients. ^2^ Comparison between normal weight and overweight patients. ^3^ Comparison between normal weight and overweight/obese patients.

**Table 3:** Glycaemic indices.

	Total Population	Normal Weight	Overweight	Obese	Overweight/ Obese	*p* Value ^1^	*p* Value ^2^	*p* Value ^3^
**HbA1c (%)**	7.2 (5.6–10)	7.2 (6.1–10)	7.4 ± 1.0	7.0 ± 0.6	7.2 (5.6–9.7)	0.106	0.468	0.185
**GMI (%)**	6.9 (5.7–8.9)	6.9 (6.0–8.9)	6.9 (6.3–8.8)	6.7 ± 0.4	6.8 (5.7–8.8)	0.072	0.768	0.261
**CV (%)**	39.5 ± 6.4	39.7 ± 7.4	40.1 ± 5.0	37.8 ± 6.0	39.2 ± 5.4	0.406	0.839	0.760
**Mean glucose (mg/dL)**	148 (101–235)	151 (113–235)	148 (125–228)	142 ± 18	145 (101–228)	0.063	0.677	0.220
**TIR (%)**	66 (25–94)	64 ± 16	63 (32–81)	69 ± 10	65 (32–83)	0.394	0.621	0.943
**TBR_70_ (%)**	4 (0–16)	4 ± 3	4 (0–16)	4 (1–16)	4 (0–16)	0.305	0.479	0.309
**TBR_54_ (%)**	1 (0–11)	1 (0–8)	0.5 (0–5)	1 (0–11)	1 (0–11)	0.304	0.748	0.458
**TAR_180_ (%)**	20 ± 7	20 ± 7	22 ± 7	19 ± 7	21 ± 7	0.518	0.401	0.788
**TAR_250_ (%)**	6 (0–40)	7 (0–40)	7 (1–37)	5 ± 4	5 (0–37)	0.026	0.699	0.165

**Abbreviations:** CV: coefficient of variation; GMI: glucose management indicator; HbA1c: glycated haemoglobin; TAR: time above range; TBR: time below range; TIR: time in range. **Notes:** Categorical variables are expressed as numbers (percentages), and continuous variables with or without normal distribution are expressed as mean ± standard deviation or median (range), respectively. ^1^ Comparison between normal weight and obese patients. ^2^ Comparison between normal weight and overweight patients. ^3^ Comparison between normal weight and overweight/obese patients.

**Table 4:** Achievement (%) of optimal glycaemic targets.

	Total Population	Normal Weight	Overweight	Obese	Overweight/ Obese	*p* Value ^1^	*p* Value ^2^	*p* Value ^3^
**HbA1c < 7%**	26 (36%)	11 (31%)	9 (38%)	6 (43%)	15 (39%)	0.448	0.628	0.473
**GMI < 7%**	41 (57%)	18 (51%)	13 (57%)	10 (71%)	23 (62%)	0.201	0.704	0.358
**CV ≤ 36%**	25 (34%)	12 (34%)	6 (25%)	7 (50%)	13 (34%)	0.308	0.447	0.995
**TIR > 70%**	22 (30%)	12 (34%)	5 (21%)	5 (36%)	10 (26%)	0.924	0.262	0.458
**TBR_70_ < 4%**	28 (38%)	15 (43%)	8 (33%)	5 (36%)	13 (34%)	0.646	0.461	0.448
**TBR_54_ < 1%**	34 (47%)	17 (49%)	12 (50%)	5 (36%)	17 (45%)	0.414	0.914	0.743
**TAR_180_ < 25%**	55 (75%)	26 (74%)	17 (71%)	12 (86%)	29 (76%)	0.386	0.770	0.841
**TAR_250_ < 5%**	26 (36%)	9 (26%)	10 (42%)	7 (50%)	17 (45%)	0.101	0.198	0.090

**Abbreviations:** CV: coefficient of variation; GMI: glucose management indicator; HbA1c: glycated haemoglobin; TAR: time above range; TBR: time below range; TIR: time in range. **Notes:** Categorical variables are expressed as numbers (percentages). ^1^ Comparison between normal weight and obese patients. ^2^ Comparison between normal weight and overweight patients. ^3^ Comparison between normal weight and overweight/obese patients.

**Table 5:** Correlations between BMI, body weight, waist circumference, and glycaemic indices in total population.

	BMI		Body Weight		Waist Circumference	
	r	*p* Value	r	*p* Value	R	*p* Value
**HbA1c**	−0.185	0.118	−0.148	0.212	−0.169	0.156
**GMI**	−0.227	0.049	−0.225	0.049	−0.109	0.365
**CV**	−0.044	0.713	−0.122	0.303	−0.148	0.215
**Mean glucose**	−0.231	0.049	−0.225	0.049	−0.113	0.345
**TIR**	0.132	0.264	0.181	0.126	0.118	0.324
**TBR_70_**	0.124	0.294	0.068	0.568	−0.004	0.973
**TBR_54_**	0.069	0.561	0.032	0.790	−0.011	0.930
**TAR_180_**	−0.124	0.294	−0.133	0.261	−0.018	0.883
**TAR_250_**	−0.251	0.033	−0.252	0.032	−0.180	0.131

**Abbreviations:** BMI: body mass index; CV: coefficient of variation; GMI: glucose management indicator; HbA1c: glycated haemoglobin; r: correlation coefficient; TAR: time above range; TBR: time below range; TIR: time in range.

**Table 6:** Effect of BMI, body weight, and waist circumference on glycaemic indices in total population.

	BMI			Body Weight			Waist Circumference		
	β	SE	*p* Value	β	SE	*p* Value	β	SE	*p* Value
**HbA1c**	−0.038	0.021	0.072	−0.009	0.007	0.197	−0.009	0.007	0.234
**GMI**	−0.035	0.014	0.012	−0.010	0.005	0.035	−0.008	0.005	0.124
**CV**	−0.162	0.142	0.259	−0.059	0.047	0.215	−0.076	0.049	0.122
**Mean glucose**	−1.517	0.562	0.009	−0.427	0.190	0.028	−0.329	0.200	0.105
**TIR**	0.636	0.302	0.039	0.188	0.101	0.067	0.151	0.106	0.159
**TBR_70_**	0.117	0.076	0.126	0.021	0.025	0.414	0.004	0.027	0.873
**TBR_54_**	0.072	0.045	0.115	0.018	0.015	0.248	0.015	0.016	0.330
**TAR_180_**	−0.302	0.149	0.047	−0.068	0.050	0.179	−0.025	0.053	0.634
**TAR_250_**	−0.516	0.205	0.014	−0.155	0.069	0.027	−0.142	0.072	0.049

**Abbreviations:** β: regression coefficient; BMI: body mass index; CV: coefficient of variation; GMI: glucose management indicator; HbA1c: glycated haemoglobin; SE: standard error; TAR: time above range; TBR: time below range; TIR: time in range.

## Data Availability

The raw data supporting the conclusions of this article are available by the corresponding author upon request.
